# Investigation and Validation of New Heart Rate Measurement Sites for Wearable Technologies

**DOI:** 10.3390/s25072069

**Published:** 2025-03-26

**Authors:** Jumana Matouq, Ibrahim AlSaaideh, Oula Hatahet, Peter P. Pott

**Affiliations:** 1 Department of Biomedical Engineering, School of Applied Medical Sciences, German Jordanian University, Amman 11180, Jordan; i.alsaaideh@gju.edu.jo (I.A.);; 2 Institute of Medical Device Technology, University of Stuttgart, 70569 Stuttgart, Germany

**Keywords:** heart rate, foot-wearables, PPG, SDG 3, dorsalis pedis artery, posterior tibial artery

## Abstract

A recent focus has been on developing wearable health solutions that allow users to seamlessly track their health metrics during their daily activities, providing convenient and continuous access to vital physiological data. This work investigates a heart rate (HR) monitoring system and compares the HR measurement from two potential sites for foot wearable technologies. The proposed system used a commercially available photoplethysmography sensor (PPG), microcontroller, Bluetooth module, and mobile phone application. HR measurements were obtained from two anatomical sites, i.e., the dorsalis pedis artery (DPA) and the posterior tibial artery (PTA), and compared to readings from the Apple Smartwatch during standing and walking tasks. The system was validated on twenty healthy volunteers, employing ANOVA and Bland-Altman analysis to assess the accuracy and consistency of the HR measurements. During the standing test, the Bland-Altman analysis showed a mean difference of 0.08 bpm for the DPA compared to a smaller mean difference of 0.069 bpm for the PTA. On the other hand, the walking test showed a mean difference of 0.255 bpm and −0.06 bpm for the DPA and PTA, respectively. These results showed a high level of agreement between the HR measurements collected at the foot with the smartwatch measurements, with superiority for the HR measurements collected at the PTA.

## 1. Introduction

Wearable healthcare solutions provide significant advantages compared to traditional methods, allowing seamless health tracking during daily activities. These solutions provide convenient and continuous access to vital physiological data, benefiting both clinical and non-clinical populations through early detection and improved patient outcomes [1,2]. The global market size of wearable technology is expected to grow between 2024 and 2030 at a compound annual growth rate of 13.6% [3]. Also, wearable technologies, including smartwatches and heart rate (HR) monitors, have been identified as the #1 fitness trend for 2024, according to a survey by the American College of Sports Medicine (ACSM) [4]. This highlights the growing use of wearables, which advancements in sensor technology, low-power and high-speed computing technologies, and communication could drive [5].

Research in wearable healthcare solutions has long focused on wearable wrist devices such as smartwatches, which can monitor various physiological parameters such as heart rate, physical activity, blood pressure, sleep position, and respiratory rate [6,7]. While initial efforts concentrated on wrist and finger wearables due to their accessibility and ease of use, different studies have expanded the scope to explore whole-body sites for health monitoring, such as the chest [8], head [9], earlobe [10], waist [9], and feet [11]. Among these alternative sites, foot-based health monitoring systems have received particularly special attention as they provide a location for tracking an individual’s overall health while being unnoticeable and natural [9]. Additionally, physiological and biomechanical signs recorded from the foot, such as muscle activity, gait parameters, arterial oxygen saturation, temperature, and humidity, could offer critical insights into various neurological and peripheral vascular health conditions [11,12,13,14]. Measuring the HR from the foot is also beneficial for specific medical conditions where wrist monitoring is impaired, i.e., edema, inflammation, upper-limb amputations, and in Parkinson’s disease, in which the foot readings can be more stable than wrist measurements affected by tremors. Furthermore, these systems provide a solution for certain sports players who are prohibited from wearing watches, bracelets, fitness trackers, or any hand wearables during matches for safety concerns.

Existing studies highlight the potential of foot-based monitoring but also reveal limitations. For instance, Zhang et al. [9] developed “FeetBeat”, a shoe-integrated device to measure muscle activity and HR from the dorsal pedal pulse using an array of pressure sensors, although the recording of the vital signs was limited to the stationary body state. Similarly, Hong and Park [15] analyzed different sensor positioning on the foot sole to measure HR in standing individuals. Datcu et al. [16] developed an innovative pulse oximeter sock for newborns, which measures HR and blood oxygen saturation for newborns. In another research, a foot-worn photoplethysmography (PPG) sensor was used for energy harvesting and HR estimation [17]. However, this was limited by motion artifacts, emphasizing the need for improved algorithms for practical and reliable clinical use [5]. Other researchers measured HR using an insole with piezoelectric films, but the signals were weak until the participants exercised to increase cardiac output and enhance the signals [5,9,18]. Jarchi and Casson [17] used signal processing to overcome motion artifacts in estimating HR using a PPG sensor during high-intensity cycling, while others deployed deep learning algorithms to predict HR from multiple sensors placed inside a smart shoe during different activities [19,20].

Despite advancements in foot-based healthcare wearables, there remain several unexplored measurement sites and sensors that could provide valuable insights for enhancing the effectiveness and accuracy of wearable health technology. This work aims to (1) investigate and evaluate the performance of a PPG-based system in monitoring HR from the foot in a stationary position as well as while walking and (2) test the feasibility of using PPG technology to measure HR from two distinct anatomical sites on foot, i.e., posterior tibial artery (PTA) and dorsalis pedis artery (DPA), and identify the optimum site, which is defined quantitatively as minimal deviation from a reference HR measurement at the chosen body location. The proposed system is ultimately intended for future integration into a low-cost, non-intrusive foot wearable for monitoring HR, vitals, and mobility in athletes and individuals with cardiovascular or metabolic conditions, though this lies beyond the scope of the current work. This integration, in return, ensures healthy lives and promotes well-being for all at all ages, aligning with the objectives of Sustainable Development Goal 3 (SDG 3): “Good health and well-being” [21].

## 2. Materials and Methods

**Figure 1:**
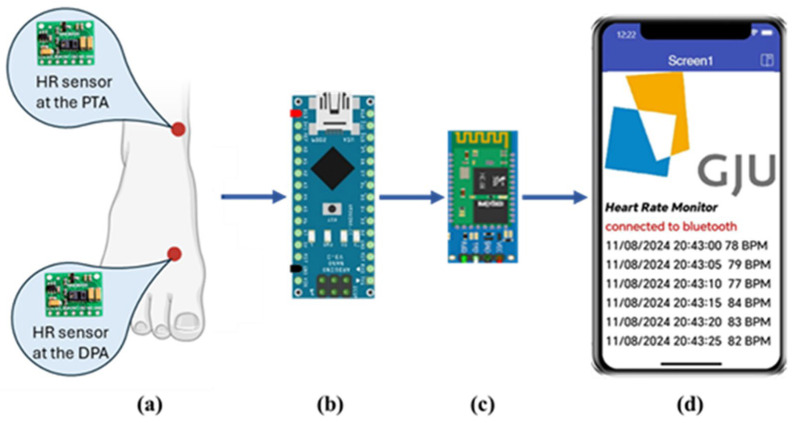
Block diagram of the proposed system. (**a**) location of the HR sensors at the DPA and the PTA. (**b**) the Arduino Nano microcontroller. (**c**) HC06 Bluetooth module. (**d**) a screenshot of the HR monitoring app developed using the MIT App Inventor.

The block diagram of the proposed system is shown in Figure 1. It consists mainly of the PPG sensor, microcontroller, Bluetooth module, and mobile phone application. Each of these parts will be explained in the following sections.

### 2.1. Sensor Location

The arterial pulse provides valuable cardiovascular insights that can be used to monitor and assess both overall health and cardiovascular diseases [22,23]. These arterial pulses can be measured in different sites in the body, including the carotid artery, brachial, femoral, popliteal, posterior tibial, and dorsalis pedis [22]. In the foot, DPA and PTA are easily palpable pulses in healthy individuals [24] and are commonly assessed in clinical settings using surface palpation [25]. Both arteries are vital peripheral pulses to assess pulse rate, quality, and rhythm, which are important in detecting vascular issues [22].

### 2.2. Photoplethysmography (PPG) Sensor

The PPG-based sensor MAX30100 (Analog Devices, Wilmington, MA, USA) was used in this work. It is an integrated pulse oximetry and HR module that combines a photodetector, two LEDSs, optimized optics, fast data output capabilities, and low-noise analog signal processing to detect pulse oximetry and HR signals [26]. Several studies have used and validated the PPG sensor MAX30100 (Analog Devices, Wilmington, MA, USA) for pulse oximetry and HR monitoring applications [5,15,16,17,27,28,29]. This sensor has many advantages, such as minimum power consumption to increase battery life for wearable applications, resilience to motion artifacts due to the high signal-to-noise ratio (SNR), and a built-in ambient light cancellation to improve its accuracy [26].

### 2.3. Microcontroller

In this work, an Arduino Nano microcontroller (Arduino LLC, Somerville, MA, USA) was used, which is one of the smallest Arduino boards, i.e., dimensions of 43 × 18 × 19 mm. The Arduino Nano microcontroller is based on the Atmega328 microcontroller. It was used in this study to program and automate the control of the sensors and transmit the data recorded by the sensor to the Bluetooth module through the transceiver pins. The PPG sensor MAX30100 (Analog Devices, Wilmington, MA, USA) was connected to the microcontroller using the Inter-Integrated Circuit (I^2^C) interface, with appropriate voltage level adjustments and pull-up resistors to ensure reliable communication. The Arduino Nano microcontroller was powered using a 9 V battery.

### 2.4. Bluetooth Module

The HC06 Bluetooth module was used to transmit the data wirelessly to the user’s mobile phone using the Universal Synchronous/Asynchronous Receiver/Transmitter (UART) protocol. This module has the advantages of ease of communication and interfacing, power efficiency, and cost efficiency, thus making it well-suited for integration into portable applications.

### 2.5. Mobile Application

A mobile phone application was developed using the MIT App Inventor (Version nb198, Massachusetts Institute of Technology, Cambridge, MA, USA), which is an online development platform for building mobile phone applications using a visual programming language for both Android and iOS operating systems [30]. This application was developed to provide a user interface for sharing the HR data recorded by the PPG sensor with the user’s mobile phone from the best-performing site of the two proposed sites, i.e., DPA and PTA, see Figure 1d.

### 2.6. Data Acquisition

HR data was collected using two PPG sensors simultaneously, each connected to a microcontroller. These sensors were placed at the DPA and the PTA, as shown in Figure 1a. The PPG sensor was calibrated before the experiment according to manufacturer guidelines to ensure accurate readings. The Apple Smartwatch series 5 (Apple Inc., Cupertino, CA, USA) was placed on the participant’s wrist as a reference for HR measurement. Once powered, the PPG sensor uses photodiodes to convert reflected light into an electrical signal, filters out 50 Hz/60 Hz interference and low-frequency ambient noise, and then digitizes the signal using its integrated 16-bit analog-to-digital converter (ADC), eliminating the need for an external ADC module [26].

After that, the resulting signal is transferred to the microcontroller, where the DC offset is removed, and a peak detection algorithm is then applied to calculate the HR in beats per minute (BPM) by measuring the time intervals between consecutive heartbeats (or peaks) in the filtered PPG signal. To minimize potential clock drift, both controllers of the MAX30100 (Analog Devices, Wilmington, MA, USA) sensors were connected to the same PC, which ensured that the data collection occurred in a controlled and synchronized environment. The Apple Smartwatch (Apple Inc., Cupertino, CA, USA) data was obtained through its application and then exported to the same PC. Additionally, the timestamps were matched to ensure accurate synchronization during data acquisition. The PPG sensor’s fast data output capability enables precise, real-time HR monitoring with minimal delay, making it ideal for wearables. While minor delays due to signal processing might exist, they fall within acceptable limits, and the 10-minute measurement periods ensure steady HR output, minimizing the impact of fluctuations.

### 2.7. Testing

Twenty healthy subjects (10 males and 10 females, aged 18–25) volunteered to test the proposed system. Table 1 shows the participant demographic data. The volunteers were young adults without any psychological or neurological disorders. They were informed about the study’s protocol before performing the experiment and assured of their right to withdraw at any time without facing any consequences. All participants provided informed consent before their participation. This research was conducted following the guidelines of the Declaration of Helsinki and approved by the Research Ethics Review Committee of Al-Hussein Bin Talal University (IRB 2024/02).

For each participant, the PPG sensors were placed at the DP and PT arteries of their foot while wearing the Apple Smartwatch (Apple Inc., Cupertino, CA, USA) on their wrist. They were then asked to standstill for 10 minutes, followed by 10 minutes of walking at their preferred base. Meanwhile, HR was simultaneously recorded by the Apple Smartwatch (Apple Inc., Cupertino, CA, USA) and the PPG sensors.

Apple Smartwatch (Apple Inc., Cupertino, CA, USA) is considered a reliable reference for validating HR wearables, offering practicality and compatibility in real-world scenarios. Extensive studies have validated the Apple Smartwatch (Apple Inc., Cupertino, CA, USA) for HR measurement with standard measuring devices, demonstrating its high accuracy and reliability in different activities [31,32,33,34,35]. Alnasser et al. [32] found no significant differences in the ECG characteristics between the Apple Watch and the 12-lead ECG with a strong positive correlation between HR measurements from both devices. HR measurements recorded by the watch showed a strong correlation across activity levels of 0.97 during walking, 0.93 during jogging, and 0.81 during running [34]. In clinical populations, statistical plots demonstrated a good correlation without systematic error when comparing the HR measurements from the Apple Watch with those from the ECG [35].

### 2.8. Data Analysis

Variables of interest were found to follow a normal distribution as indicated by the Shapiro-Wilk test [36]. Descriptive statistics were calculated for each condition at the three sites. A two-way analysis of variance (ANOVA) was conducted to evaluate the main effects of the measurement site, i.e., wrist, DPA, and PTA, and the activity state, i.e., standing and walking, on the HR measurements. In case of significance, a post-hoc analysis was performed using Tukey’s Honest Significant Difference (HSD). Lastly, Bland-Altman analysis was performed to evaluate the mean difference and precision of the proposed system compared to the ground truth measurements of the Apple Smartwatch. The Bland-Altman analysis was performed on mean HR values derived from the entire 10-minute measurement for each subject in each activity. In the Bland-Altman test, bias represents the mean difference between the two methods, while precision is represented by the limits of agreement (LoA), which are calculated as the mean difference ±1.96 times the standard deviation of the differences [37,38]. The results are visually presented in a scatter plot, where the difference between the two measurements is represented on the y-axis, their average on the x-axis, and significance was set at *p* < 0.05. Statistical analysis was performed in R studio (Version 2024.04.0, Posit PBC, Boston, MA, USA).

## 3. Results

Table 2 summarizes the HR measured by the Apple Smartwatch and the PPG sensors at the DPA and PTA at the two activity conditions i.e., standing and walking. The ANOVA model showed no significant effect on the measurement site (*p* > 0.1). The activity state was found to have a significant effect on the HR readings obtained from the three sites (*p* < 0.01). The mean HR during standing was 77.6±0.3 bpm measured using the Apple Smartwatch and 77.6 ± 0.4 bpm measured by the PPG sensors at both the DPA and the PTA. In the walking task, mean HR values increased to 97.4 ± 0.6 bpm, measured using the Apple Smartwatch, and 97.2 ± 0.6 bpm and 97.5 ± 0.7 bpm as measured by the PPG sensors at the DPA and the PTA, respectively.

**Figure 2:**
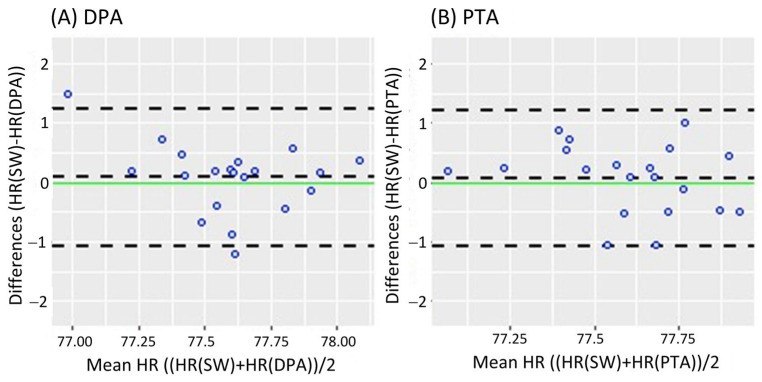
Bland-Altman plots while standing, where each data point represents the mean HR recorded per subject. (**A**) Bland-Altman plots of the HR measurements of the DPA compared to the Apple Smartwatch measurements; (**B**) Bland-Altman plots of the HR measurements of the PTA compared to the Apple Smartwatch measurements. The central horizontal dashed line shows the mean of the differences (=bias) between the two methods, and the other two lines show the upper and lower 95% limits of agreement (=bias ± 1.96 × SD). HR: Heart Rate, SW: Apple Smartwatch, DPA: Dorsalis Pedis Artery, and PTA: Posterior Tibial Artery.

**Figure 3:**
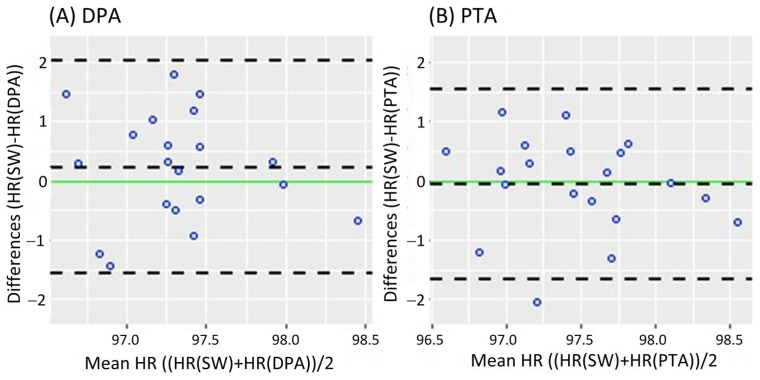
Bland-Altman plots while walking, where each data point represents the mean HR recorded per subject. (**A**) of the HR measurements of the DPA compared to the Apple Smartwatch measurements; (**B**) Bland-Altman plots of the HR measurements of the PTA compared to the Apple Smartwatch measurements. The central horizontal dashed line shows the mean of the differences (=bias) between the two methods, and the other two lines show the upper and lower 95% limits of agreement (=bias ± 1.96 × SD). HR: Heart Rate, SW: Apple Smartwatch, DPA: Dorsalis Pedis Artery, and PTA: Posterior Tibial Artery.

Figure 2 shows the Bland-Altman analysis while standing. It revealed a mean difference of 0.08 bpm (95% CI [−0.182 0.343]) and LoA of −1.079 and 1.239 bpm for the DPA (Figure 2A); meanwhile, a smaller mean difference of 0.069 bpm (95% CI [−0.191 0.329]) and narrower LoA of −1.079 and 1.217 bpm were found for the PTA, as shown in Figure 2B. On the other hand, the Bland-Altman analysis results in the walking task are shown in Figure 3. Similar to the standing task, the mean difference decreased from 0.255 bpm (95% CI [−0.181 0.631]) for the DPA to −0.06 bpm (95% CI [−0.420 0.299]) for the PTA. The LoA followed a similar pattern with −1.522 and 1.972 for the DPA (Figure 3A), which narrowed to −1.610 and 1.49 for the PTA, see Figure 3B.

## 4. Discussion

According to the author’s knowledge, this study showed for the first time that PPG sensors can be used to non-invasively measure HR from the foot at different sites with reasonable accuracy and precision. The results indicate that the readings from the PPG sensor were closely comparable at both DPA and PTA to those obtained from the Apple Smartwatch, which is very promising for wearable and healthcare monitoring applications.

The PPG sensor MAX30100 has been validated previously for measuring HR from different sites, i.e., forehead and temporal bone, with a pulse oximeter used as the reference device [39]. Datcu et al. [16] redesigned the traditional pulse oximeter into an innovative sock for newborns using the PPG sensor MAX30100, which measures HR and blood oxygen saturation. However, the testing was limited and performed on a fingertip rather than on the intended application area, and a mobile phone application for data visualization was not included. PPG sensor MAX30100 has also been integrated into different healthcare applications [40,41,42]. However, all these studies focused on extracting vital signs from the upper limbs. The consistency of the HR measured by this sensor from the foot arteries is demonstrated with the reference measurements, as well as its sensitivity to changes in the participants’ activity levels, as indicated by the results of the ANOVA test, as shown in Table 2.

Previous studies indicated that ±5 bpm or ±10% is an acceptable error range for HR measurement devices [43,44]. Bland-Altman analysis is recognized as the standard for evaluating agreement between a new measurement technique to an established one [37]. Accordingly, the Bland-Altman analysis of the proposed system resulted in small mean differences (less than 1%) and narrow LoA, indicating a high level of agreement between the proposed system and the Apple Smartwatch. Notably, the HR measured at the PTA showed superior precision and less error compared to the measurements at the DPA, with smaller mean differences and narrower LoA observed at the PTA across both activity conditions. It has been reported previously that the DPA pulse is undetectable in 3.1–13.8% of young adults, while the PTA s only 0–2.6% of this population [45]. Additionally, the DPA is often located aberrantly, and its pulse is missing at birth in approximately 10% of individuals [46].

It is worth noting that the PPG sensor MAX30100 had a smaller absolute error and higher precision when participants were standing still compared to when walking, which could be caused by motion artifacts. This finding has been reported previously for different wearable applications [33]. In this study, the recorded readings using the PPG sensor MAX30100 remained within acceptable ranges, indicating reliable performance across different activity states. It is also worth noting that the absolute error at the DPA increased more notably while walking compared to the PTA, supporting the evidence that the PTA provides a more reliable site for acquiring HR.

Although in this study, we evaluated the system performance on low-intensity movements, i.e., during standing and walking, assessing the system’s performance during higher-intensity activities remains essential, i.e., jogging and running. Such activities may introduce greater motion artifacts due to foot impact and soft tissue movement. Thus future investigations are required to assess system performance under a wider range of physical activities with various environmental conditions, including scenarios where sweating may occur.

## 5. Conclusions

This study demonstrated the validity of HR measurements from the foot using the MAX30100 PPG sensor. In addition, a comparison of two sites, i.e., DPA and PTA, showed superior performance of the PTA. These results have significant implications for wearable healthcare telemonitoring applications, as they suggest that peripheral sites such as the PTA can be effectively used for continuous and unobtrusive HR monitoring. It is important to note that this work has some limitations. First, the included age range was limited. While this is useful to control age-related variability in HR, it limits the generalizability of the findings to broader populations. Future work will focus on reducing the size of the proposed system by incorporating more compact sensors, exploring advanced sensor technologies, and investigating more efficient power solutions to enhance battery life. Additionally, the final prototype will be tested on a larger sample with a broader age range to improve generalizability. It is also important to note that previous research has shown that inaccurate PPG HR readings are up to 15% more common in darker skin tones. The sensitivity of the PPG sensor MAX30100 to skin tone has not been tested before; hence, this should be taken into account before implementing this prototype into wearables applications. The primary goal of the upcoming work will be to integrate the proposed system into wearable footwear to collect HR data from the PTA, along with other physiological signs, while incorporating design features that can guide the user to the optimal placement for improved accuracy and signal reliability.

## Figures and Tables

**Table 1:** Participant demographic data.

Variable	n = 20 (10 Females, 10 Males)
	Mean (SD)
Age (years)	21.5 (2.4)
Weight (kg)	63.4 (8.2)
Height (cm)	170.5 (6.7)
BMI	21.778 (2.3)

**Table 2:** Mean and standard deviation (SD) of participants’ HR measurements recorded by the Apple Smartwatch and the PPG sensors at the DPA and the PTA in both standing and walking conditions.

	Standing Mean (SD)	Walking Mean (SD)
Apple Smartwatch (bpm) *	77.6 (0.3)	97.4 (0.6)
Dorsalis Pedis Artery (DPA) (bpm) *	77.6 (0.4)	97.2 (0.6)
Posterior Tibial Artery (TPA) (bpm) *	77.6 (0.4)	97.5 (0.7)

* Indicates a significant difference resulting from the ANOVA model.

## Data Availability

Data is contained within the article.
